# Hydraulically informed graph theoretic measure of link criticality for the resilience analysis of water distribution networks

**DOI:** 10.1007/s41109-018-0079-y

**Published:** 2018-08-13

**Authors:** Aly-Joy Ulusoy, Ivan Stoianov, Aurelie Chazerain

**Affiliations:** 1 InfraSense Labs, Dept. of Civil and Environmental Eng., Imperial College London, Imperial College Road, London, SW7 2BU UK; 2 SUEZ environnement SAS, 38, Rue du President Wilson, Le Pecq, 78 230 France

**Keywords:** Water distribution networks, Resilience, Graph theory, Random walk betweenness centrality

## Abstract

Water Distribution Networks (WDN) are complex and highly interconnected systems. To maintain operation under failure conditions, WDNs should have built-in resilience based on topological and energy redundancy. There are various methods for analysing the resilience of WDNs based on either hydraulic models or surrogate network measures; however, not a single universally accepted method exists. Hydraulic modeling of disruptive operational scenarios suffer from combinatorial restrictions and uncertainties. Methods that rely on surrogate network measures do not take into account the complex interactions between topological and energy redundancy. To bridge this gap, the presented work introduces a hydraulically informed surrogate measure of pipe criticality for the resilience analysis of WDNs, called Water Flow Edge Betweenness Centrality (WFEBC). The WFEBC combines the random walk betweenness centrality with hydraulic (energy) loss principles in pipes. The proposed network resilience estimation method is applied to a case study network and an operational network. Furthermore, a network decomposition approach is proposed to complement the network estimation method and facilitate its scalability to large operational networks. The described resilience analysis method is benchmarked against a hydraulic model-based analysis of WDN reserve capacity. WFEBC is also applied to assess the improvement in resilience allowed by the implementation of a dynamically adaptive topology in an operational network. The benefits and limitations of the proposed method are discussed.

## Introduction

In March 2018, a series of pipe bursts left thousands of homes across the UK without access to potable water. The supply interruptions escalated over a period of a few days due to a 4,000% increase in pipe bursts in some areas (according to Severn Trent), combined with a 20% increase in domestic consumption (as reported by Thames Water) (BBC 2018). The wide spread failures to meet minimum serviceability levels were a consequence of the low resilience of water distribution networks. Extensive sectorisation of WDNs into single-feed sectors (called District Metered Areas, or DMAs) as well as aggressive pressure reduction schemes have diminished their topological and energy redundancy. These recent events illustrate the importance of resilience assessment in the design (Jayaram and Srinivasan 2008), rehabilitation, management and operational control of water distribution networks.

Design principles and pipe rehabilitation strategies in WDNs are generally based upon assessment of the vulnerability (or hydraulic reliability) of a network to specific hazards. The risk assessment is based on probabilistic measures of the consequences of failure events on the level of service of customers (Ostfeld and Shamir 1993; Xu and Goulter 1999; Deuerlein et al. 2009; Shuang et al. 2014; Diao et al. 2016). Due to the unpredictable nature (and causes) of disruptive events (Gao et al. 2016; Hosseini et al. 2016) and lack of universally accepted probabilistic approach to component (e.g. pipe, valve, pump) failures (Andreou et al. 1987; Goulter and Bouchart 1990), failure predictions in WDNs are highly uncertain. More robust measures for network design and rehabilitation strategies are based on network resilience, i.e. the ability of networks to anticipate, absorb, adapt to and/or rapidly recover from a disruptive event (Laprie 2005; Strigini 2012; Gao et al. 2016).

The resilient operation of complex networks depends on their structure (connectivity), i.e., the existence of redundant paths between pairs of nodes (vertices). Based on the study of percolation theory, the resilience analysis of complex networks is quantified by indicators such as node degree distribution (Newman 2003b) and connectivity redundancy (Albert et al. 2001). Resilience analyses are commonly applied for the investigation of the Internet (Cohen et al. 2000), the World Wide Web (Albert et al. 2001), social networks (Callaway et al. 2000), transportation networks (Crucitti et al. 2004) and electrical networks (Arianos et al. 2009). In the case of WDNs, the operation of a network also depends on the available hydraulic head (energy) and energy losses. The range of failure and threat scenarios for water distribution networks is extremely broad. It includes individual and combined pipe failures, which affect the connectivity of a network, pump failures and extreme demand conditions (e.g. fire flow). As a result, definitions and measures of resilience differ (Herrera and Abraham 2016). The UK Government and Ofwat recommend that the resilience of WDNs (and any critical infrastructure) should be secured by improving the response capacity of operators on one hand and the design of networks on the other, to ensure they are resistant to damage, designed to operate under a range of conditions and sufficiently redundant (U.K. Government Cabinet Office 2011). There are, however, no formal quantitative metrics for these activities and components that can be included in the design and operation of water distribution networks.

Building resilience within complex and interconnected water distribution networks in order to provide continuous services requires the identification and mapping of critical links (pipes) that could lead to disruption of services. Consequently, the work presented in this paper focuses on analysing the criticality of links (pipes) towards the overall resilience of a network by combining graph theory methods and basic principles of pipe hydraulics. It builds upon previous studies of WDN resilience based on surrogate measures of spare capacity, in the form of energy or topological redundancy. As discussed in the background review section, traditional hydraulic methods suffer combinatorial restrictions as well as limitations due to the uncertainty of the hydraulic models. Most surrogate measures of redundancy on the other hand overlook the complex interaction between topology and energy redundancy (Di Nardo et al. 2017).

In order to address these limitations, a hydraulically informed graph theoretic measure of pipe criticality is proposed in this paper, called Water Flow Edge Betweenness Centrality (WFEBC). WFEBC is derived from random walk betweenness centrality for the resilience analysis of complex WDNs and the estimation of energy losses. WFEBC is applied to two case study networks. The proposed analytical framework includes a network skeletonisation step, in the form of a forest-core decomposition of a network, in order to facilitate the application of the proposed resilience estimation to large scale operational networks. The surrogate measure is benchmarked against a traditional hydraulic analysis. The results show that WFEBC provides unique insights into the criticality of individual links and identifies low resilience sectors of a system for further hydraulic analysis. It is applied to compare the resilience of a sectorised operational network (single-feed DMAs) to the resilience of the same network following the implementation of a dynamically adaptive topology (multi-feed DMAs). Finally, a summary of the results is presented together with suggestions about the applicability and improvements of the proposed method.

## A review of WDN resilience and critical component analysis

Aging infrastructures, investment constraints (Wagner et al. 1988) and increasing regulatory pressures make resilience a key decisional criterion for optimising the capital investment in pipe rehabilitation and risk mitigation strategies. Operators rely on critical link analyses (CLA) to understand the contribution of individual pipes to the overall resilience of a network. Measures of link criticality are either derived from a hydraulic model of a network or from surrogate measures of energy and/or topological redundancy. The following sub-sections summarise the two approaches.

### Hydraulic model based approach for CLA

The resilience of a network to a defined set of failure scenarios can be assessed by hydraulically simulating their consequences on the performance of a network (Todini 2000; Wright et al. 2015). A resulting deficiency in meeting the nominal demand of customers and partial failure states measures the impact of a corresponding failure scenario.

**Fig. 1:**
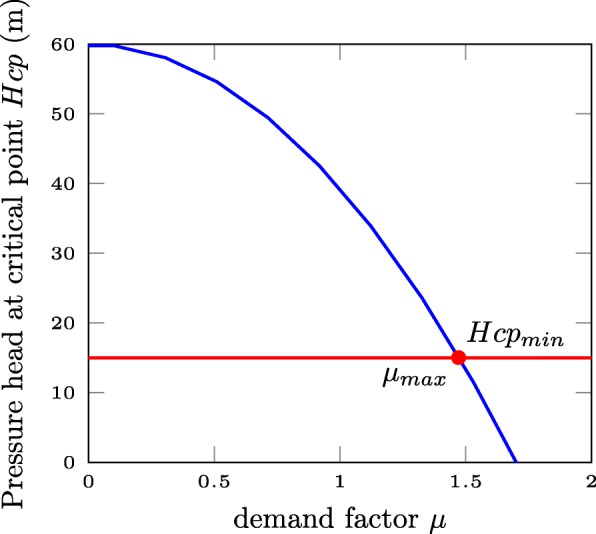
Definition of reserve capacity. As the demand factor *μ* increases, the pressure head at the critical node decreases, until it reaches the minimum allowed pressure. The value of *μ* when the pressure head at the critical point reaches the service head defines the reserve capacity of a network

Hydraulic simulation tools commonly rely on a demand driven analysis (DDA). A DDA assumes the design customer demand is always met, regardless of the available pressure. Consequently, they are not able to accurately replicate the behaviour of WDNs under pressure deficient conditions (Gupta and Bhave 1994). Different metrics (Shamir and Howard 1981; Wagner et al. 1988; Fujiwara and De Silva 1990; Bao and Mays 1990) are suggested to account for partial failure states according to pressure thresholds defined by Wagner et al. (1988) but the absence of dependency between demand and pressure in the demand driven analysis ultimately yields hydraulically inaccurate results. Wright et al. (2015) proposed to address the issue by running multiple demand driven analyses to identify the reserve capacity of a network, i.e. the maximum demand factor for which pressure levels are greater than the service head at all nodes. Its calculation is formulated as an optimisation problem where *μ*, the nominal demand multiplier, is maximised subject to the mass and energy balances. Figure 1, adapted from (Wright et al. 2015), illustrates the optimisation procedure that is repeated for all failure scenarios. This approach is computationally intensive, and relies on the definition of a critical point, but allows to quantify both pressure deficient and excess pressure network topologies. This makes it particularly suitable as a hydraulic model based approach for estimating the resilience of a WDN; and it was used as a benchmark for the surrogate measure developed in this study (details about the definition of reserve capacity can be found in (Wright et al. 2015)). Alternatively, Gupta and Bhave (1994) suggested replacing the demand driven analysis with a pressure driven analysis (PDA) (Bhave 1981; 1991) to account for pressure demand relationships in WDNs. Derived from field data, the empirical relationships between available pressure head and effective demand (both customer demand and leak flow) (Jowitt and Xu 1990; Vairavamoorthy and Lumbers 1998) depend on the hydraulic configuration of the network (Germanopoulos 1985) and contain a high degree of uncertainty (Lee et al. 2016). Another limitation of PDA is that energy losses resulting from failure events are in general partially compensated by built-in excess capacity. Unlike reserve capacity, measures of demand shortfall based on PDA can only reflect energy losses associated with supply shortages.

For real networks, hydraulically simulating the consequences of all failure scenarios to perform a higher-state reliability assessment on simultaneous pipe failures is combinatorially and computationally prohibitive (Berardi et al. 2015; Gheisi and Naser 2015). A more common approach consists of restricting the set of failure scenarios to individual link failures and performing a critical link analysis (CLA) to identify the importance of individual network components. Hydraulic model based CLA results provide an accurate representation of the impact of individual pipe failure scenarios on the hydraulic performance of a network. Despite the restricted set of considered failure scenarios, the CLA requires a significant computational effort for large networks as independent hydraulic simulations must be run to evaluate the impact of each individual component failure (Berardi et al. 2015). The complexity of the problem as well as the number of different failure scenarios grow exponentially with the size of a network (Deuerlein et al. 2009; Berardi et al. 2015; Herrera et al. 2015).

The limitations of hydraulic model based CLA restrict its application to the identification of critical pipes in large WDNs, which has prompted a significant interest in analytical surrogate measures of WDN resilience. Surrogate measures of redundancy or resilience based on graph theory can be used to rank and restrict the search space for critical pipes prior to further hydraulic validation (Di Nardo et al. 2018) in the following way: 1) a CLA is first carried out on a network using a graph theoretic surrogate measure of link criticality, 2) links with lowest criticality values are discarded, 3) a hydraulic CLA (based on measures of reserve capacity for instance) is performed on the remaining critical links. This analysis is summarised by Fig. 11 and later illustrated on a case study network.

### Surrogate measures of link criticality based on topological and energy redundancy

A WDN must be able to cope with a wide range of failure events, from pipe failures to sudden increases in demand (e.g. fire fighting flows) (Atkinson et al. 2014; Creaco et al. 2016). The mechanical and hydraulic reliability define the resilience of a WDN as a function of its redundant capacity, in the form of excess energy (energy redundancy) and alternative supply paths (Di Nardo et al. 2017):

- The **energy redundancy** of a WDN measures the excess pressure available, i.e. nodal pressures higher than the nominal pressure, either within the whole network or at a defined critical node. An important component of WDN resilience, energy redundancy is increasingly at risk because of leakage minimisation practices (aggressive pressure reduction and network sectorisation);
- The **topological redundancy** of a WDN depends on the availability of loops and alternative supply routes. Tree-like structures, on the other hand, provide limited options for alternative supply routes.

The greater the built-in energy and topological redundancy of a network, the greater its resistance to disruption. Many surrogate measures of global WDN resilience consider energy (Gessler and Walski 1985; Todini 2000) and topological redundancy (Freeman 1977; Buhl et al. 2006; Yazdani and Jeffrey 2010; 2011; 2012; Wright et al. 2015). The contribution of individual links to the redundancy of a network is now being increasingly used as a measure of link criticality; as early approaches find inspiration from mechanical and electrical engineering systems (Goulter and Coals 1986; Su et al. 1987; Wagner et al. 1988; Gupta and Bhave 1994).

Both energy and topological redundancy metrics have their limitations as surrogate measures for network resilience and critical link analysis. Measures of energy redundancy rely on a single simulation under normal operating conditions to derive the excess energy in the network (Creaco et al. 2016). These connectivity assumptions do not hold under failure conditions (Herrera et al. 2015; Giustolisi et al. 2008) and the resulting measures generally fail to provide connectivity insights (Atkinson et al. 2014): high levels of energy redundancy might be a result of increased topological redundancy (Wright et al. 2015) or from built-in excess pressure capacity (Raad et al. 2010). On the other hand, purely topological approaches assume that adequate operation of the network solely relies on the connectivity of a graph when in reality, the effective serviceability (reachability of nodes) depends on multiple factors such as hydraulic head and energy losses within a network. Topological analyses provide more connectivity insights compared to energy redundancy measures but they overlook the fluid mechanics of a network and, as a result, are decorrelated from hydraulic performance indicators (Torres et al. 2017). In (Wagner et al. 1988), Wagner et al. concluded that topological reachability is a necessary but not sufficient condition to ensure customer demand is met. The resilience of a water distribution system is a complex function of both its topological and energy redundancy (Di Nardo et al. 2017).

Further research combined topological and hydraulic approaches to quantify the total redundancy of a network and the contribution or criticality of individual network components (Awumah et al. 1990; Tanyimboh and Templeman 1993; Prasad and Park 2004; Setiadi et al. 2005; Prasad and Tanyimboh 2009; Raad et al. 2010). Graph theoretic measures of node criticality are weighed to address the topological uniformity of WDNs: as a result of the physical and geographic constraints affecting their topology (link density, node degree, network diameter,...), WDNs are mostly organised as homogeneous planar graphs (more specifically, *nested pseudo-random graphs* (Giudicianni et al. 2018)) and cannot be characterised with unweighted graph theoretic metrics (Yazdani and Jeffrey 2010; 2011). With their measure of *entropic degree*, Bompard et al. (2009) suggested to complement node strength by also accounting for the distribution of weights among the links (edges). An extra dimensionless weighting factor was introduced by Yazdani and Jeffrey to differentiate between types of nodes and derive the *demand-adjusted entropic degree* (Yazdani and Jeffrey 2012).

Measures of criticality based on betweenness centrality (Yazdani et al. 2011) were also investigated to assess the central-point dominance of WDN nodes. Shortest path betweenness centrality was first defined by Freeman (1977) as a measure of agent centrality in communication networks: the larger the betweenness centrality of a node, the more shortest paths it is located on and the more central it is to the flow across the network. Applying this approach to WDNs, Di Nardo and Di Natale yielded a measure of path frequency based on the number of shortest paths from source to customer node a link is featured on (Di Nardo and Di Natale 2011). In most networks however, flow is unlikely to be constrained to the paths of least resistance (Freeman et al. 1991; Newman 2005). More realistic betweenness measures are developed that account for non-geodesic paths: in their criticality analysis. Herrera et al. (2015), Herrera and Abraham (2016) extended node betweenness to the k-shortest paths of the network (Eppstein 1997). Freeman et al. (1991) suggested accounting for all independent paths between all pairs of nodes using Ford and Fulkerson’s model (Ford and Fulkerson 1956a; 1956b; 1962). This is done to determine the maximum flow through the network as it is assumed that the flow between a pair of nodes is a physical phenomenon that depends on the capacities of all paths connecting these nodes. For some processes, the assumption of ideal flow propagation is considered too unrealistic and, as a result, *random walk betweenness*, a measure based on random explorations of the network (Newman 2005; Brandes and Fleischer 2005), is developed. The principle of this approach is that flow spreads over all adjacent links proportionally to their conductance at each step of the random walk; this is similar to the flow of electrical current in a linear circuit (Doyle and Snell 1984; Newman 2003a). In this way, the approach replicates the propagation of the flow through all available pipes while prioritising shorter paths.

This paper work describes the development of a measure of WDN link criticality derived from random walk betweenness centrality that also accounts for energy losses along the pipes. The new measure, Water Flow Edge Betweenness Centrality (WFEBC), is presented in the next section.

## Water Flow Edge Betweenness Centrality

The presented research describes the development of a surrogate measure for link criticality analysis that applies random walk betweenness centrality together with physical metrics of energy losses governing the water flow in pipes. The *water flow edge betweenness centrality* (WFEBC), is a continuation of previously published research that attempted to integrate elements of energy redundancy to graph theoretic measures of centrality (Herrera et al. 2015; Herrera and Abraham 2016). Following from Newman’s random walk betweenness centrality measure, WFEBC takes an alternative approach to traditional shortest-path betweenness measures (Herrera et al. 2015) (see previous section), which constrain the flow to the geodesic paths of a network.

### Random-walk edge betweenness centrality

The motivation for investigating random walk betweenness centrality stemmed from the observation that water flows, like information, vehicles and electric current, are not, in general, constrained to ideal propagation paths (Newman 2005). This assumption is reasonable in most networks: flows tend to spread over all available paths based on their resistance. The random walk betweenness of a node or edge *i* is thus defined as the number of random walks between couples of source and target nodes *s* and *t* passing through *i*. Considering random walks instead of shortest paths allows to account for alternative paths that the latter approach would typically disregard. This results in a more accurate and realistic estimation of the criticality of the links and overall physical resilience of the network. The reader is referred to (Newman 2005) and (Brandes and Fleischer 2005) for the original definition of current flow betweenness centrality.

In social networks, nodes generally represent information holding agents. In the case of water distribution systems, it is more relevant to focus on the betweenness centrality of edges to understand the implications of the failure scenarios on the performance of the network.

### Water flow edge betweenness centrality

A main contribution of this work is the adaptation of random walk betweenness centrality to the analysis of the criticality of individual pipes with regards to their contribution to the overall resilience of a WDN. The following additions have been made to Newman’s definition of random walk betweenness centrality:

- The sets of source and target nodes, between which random walks are computed, are restricted in accordance with the operational functions of these nodes in a network, the sources of supply (reservoirs, tanks, pumping stations) and customer nodes (consumers);
- The adjacency matrix of the graph is weighted by coefficients derived from physical conservation laws governing flow dynamics in a network, to account for differences in the frictional resistance of pipes (edges);
- Similarly to the definition of Routing Betweenness Centrality (RBC) (Dolev et al. 2010), the unit supply between a given pair of source-target (s-t) nodes is weighted both by the relative demand at the target node and the relative capacity of the source node, compared respectively to the total demand and total capacity of a network.

These modifications aim to incorporate both the operational functions of nodes and the energy redundancy of a network in the graph theoretic metric for CLA. The definition and calculation procedure of WFEBC, which is derived from Newman’s current flow analogy, are outlined below.

#### Definition

The sets of source and target nodes (vertices) should be defined before the WFBEC is computed for a network. Nodes are divided into two sets, based on the operational function they have in a network. The set of sources *S* comprises the water sources for a water supply network (e.g. treatment works, pumping stations, reservoirs, tanks, …). Each source node is associated with a factor *c*_*s*_ representing the capacity of the source compared to the total source capacity for a network. The target set *T* comprises of the customer nodes of the network, associated with a factor *q*_*t*_ measuring their demand relative to the total demand of the network. The betweenness *b*_*e*_ of edge *e* is obtained by computing the weighted sum of individual betweenness values $b_{e}^{st}$ for all source-target node pairs (*s*,*t*), where *s* and *t* belong to the sets *S* and *T*. It is a special case of routing centrality; individual betweenness values $b_{e}^{st}$ are weighed by *c*_*s*_×*q*_*t*_, which corresponds to the number of sending packets from source *s* to target *t* in a communication network:

1 $$ b_{e} = \sum_{(s,t) \in (S,T)}{c_{s} q_{t} Q_{e}^{st}}   $$

where $Q_{e}^{st}$ is the fraction of unit flow from s to t flowing through edge e. The criticality of edge *e* is then obtained by normalising *b*_*e*_ by the maximum total flow *e* would have to support if no alternative path was available.

The novelty of WFEBC is the inclusion of alternative supply paths, for which shortest-path betweenness centrality measures fail to account, over-estimating the criticality of principal supply paths as a result. WFEBC gives an account of the fraction of the total demand of the network depending on the integrity (operational status) of a pipe. It is a measure of the relative criticality of alternative supply paths and an approximate estimation of the energy losses incurred by the flow redistribution following their failure. If exclusive (or single) supply paths fail, the associated flow is not redistributed among alternative paths. By definition, their WFEBC is 1.

#### Calculating WFEBC

We consider a WDN with *n*_*n*_ demand nodes, *n*_0_ sources and *n*_*p*_ pipes. The network is assimilated to a weighted graph with *n*_*n*_+*n*_0_ vertices and *n*_*p*_ edges. We define the set of *n*_0_ source nodes *S* and *n*_*n*_ target nodes *T*, the vector of flows through the network edges *Q* and the vector of potential at network nodes *V*. A weighted adjacency matrix *A* is also defined that represents the conductance of the real system pipes. A surrogate measure for energy loss is derived from the Darcy-Weisbach equation:

2 $$ A_{ij} = \left\{\begin{array}{ll} \frac{d_{ij}}{L_{ij}} & \text{if there is an edge e connecting node i and j,}\\ 0 \qquad & \text{otherwise.} \end{array}\right.   $$

where *L*_*ij*_ and *d*_*ij*_ are respectively the length and diameter of the pipe connecting i and j. Depending on the availability of hydraulic data, the definition of pipe resistance can be improved in accordance with the Darcy-Weisbach to include the pipe material or friction factor.

We adapt Newman’s (2005) current flow analogy to compute WFEBC using the weighted matrix *A* defined in Eq. 2: the propagation of a unit current flow is a linear function of the resistances of the edges of the electrical network and the resulting flow distribution is equivalent to that of an infinite random walk through the network.

We consider a unit flow from node *s*∈*S* to node *t*∈*T*, represented by vector *S*^*st*^:

3 $$ S_{i}^{st} = \left\{\begin{array}{ll} +1 &\text{for} \quad i=s,\\ -1 &\text{for} \quad i=t,\\ 0 &\text{otherwise.} \end{array}\right.  $$

Flow conservation in the network states:

4 $$ \sum_{j} A_{ij}|V_{i}-V_{j}| = S_{i}^{st}  $$

This is equivalent to

5 $$ (D-A) V = S^{st}   $$

*D* here represents the diagonal matrix of node strengths, and not degrees, since we consider the weighted adjacency matrix *A*:

6 $$ D_{ii} = \sum\limits_{j=1}^{n_{n}+n_{0}}{A_{ij}}  $$

Due to the conservation of flow in the network, the graph Laplacian *D*−*A* is singular and the solution to Eq. 5 is defined to within an arbitrary additive constant (Newman 2005). To address the issue, we can set the value of *V*_*t*_ to 0 and remove the *t*^*th*^ row and *t*^*th*^ column from matrix *D*−*A*. The resulting matrix *D*_*t*_−*A*_*t*_ is non-singular. It is inverted and the *t*^*th*^ row and column are added back in, with all values equal to zero, yielding matrix (*D*_*t*_−*A*_*t*_)^−1^. The vector of node potentials are then given by

7 $$ V = (D_{t}-A_{t})^{-1}*S^{st}  $$

Finally, we determine the flow $Q_{e}^{st}$ through edge *e* associated with a unit supply of current from *s* to *t*:

8 $$ Q_{e}^{st} = A_{ij}*|{V_{i}-V_{j}}|   $$

Note that we consider the absolute value of the difference in potential here, as considering directed flows could minimise the importance of certain pipes: the total centrality of a link is measured as the sum of its contributions to the supply of individual target nodes. For this reason, individual link betweenness must be measured independently for each source-target node pair.

Individual betweenness values are finally input back into Eq. 1 to yield *b*_*e*_. The measure *b*_*e*_ is then, for each link, normalised by the total maximum flow that the pipe would have to carry had there been no alternative supply paths. The reader is referred to (Newman 2005) for proof of and more details about the current flow analogy.

## Case studies and results

The following section presents the analysis of a case study network (Net3) and an operational network (the Bristol Water Field Lab, or BWFL). Following an optional network reduction step (that was carried out on BWFL due to the large scale of the network), WFEBC is applied to analyse the criticality of the network links. The results are compared to a hydraulic critical link analysis that calculates the reserve capacity of a system associated with individual pipe failures.

WFEBC is a measure of the relative contribution of alternative supply paths to the redundancy of the network whereas the reserve capacity associated with a link measures the maximum fraction of the demand of the critical node the network can supply following the failure of the link. Low measures of WFEBC (resp. high reserve capacity values) indicate the availability of more alternative supply paths. Single supply paths, on the other hand, have no alternative supply routes and are expected to be identified with high criticality according to both WFEBC and reserve capacity. The following subsections describe the application of WFEBC to case studies.

### Net3

The WFEBC analysis was first carried out on Net3, a case study network of 92 nodes, 5 sources or reservoirs and 119 links. The network is included as an example with the distribution of the open-source hydraulic solver software EPANET 2.0 (Rossman 2000).

**Fig. 2:**
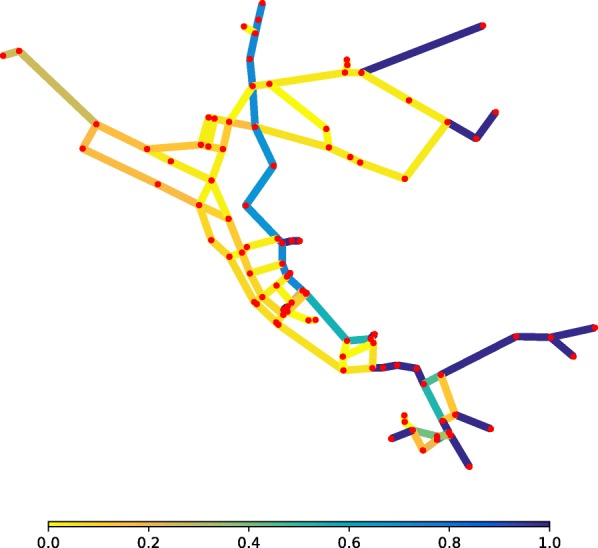
WFEBC of Net3 links. The betweenness of each link is weighted by customer demand and normalised by the maximum total flow it would have to support if there were no alternative paths to yield a measure of WFEBC. The darker the link, the more critical

WFEBC is computed for all network links as described above and the results, ranging from 0 to 1, are represented by the colours of the links on Fig. 2. The WFEBC of a link is the sum of all partial flows going through the link (as computed by the random walk for all source-target pairs of nodes) divided by the total demand of the target nodes partially supplied by the link. The greater the WFEBC of a link, the more important it is compared to alternative supply paths. A WFEBC value of 1 indicates an exclusive supply link (bridge, or bottleneck).

**Fig. 3:**
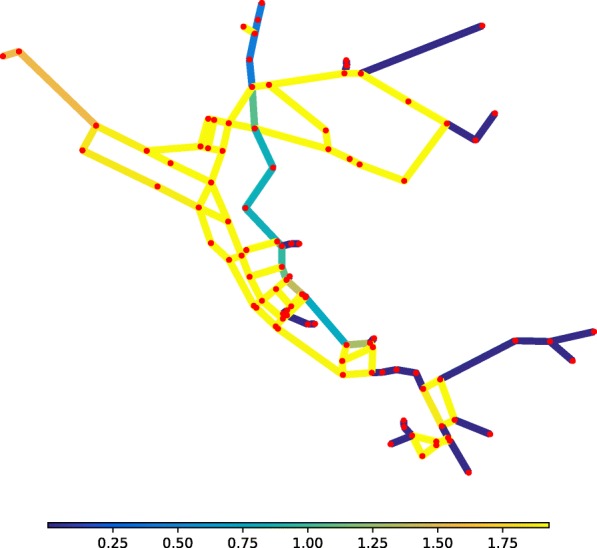
Reserve capacity of Net3 links. A hydraulic CLA is carried out to measure the reserve capacity of Net3 associated with the individual failure of each link. Reserve capacity measures the fraction of the total demand the network can supply while meeting pressure requirements. The darker the link, the more critical

The graph theoretic analysis of Net3 is compared to a hydraulic resilience analysis of the network (Fig. 3), carried out using reserve capacity (Wright et al. 2015) as an index for link criticality. In both figures, the darker the colour of the edge, the more critical it is according to the metric used.

**Fig. 4:**
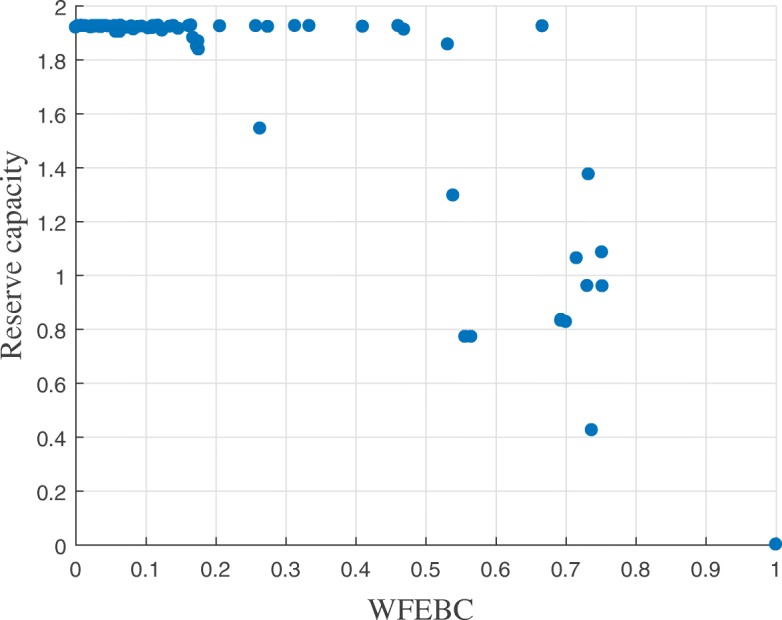
Analysis of the correlation between reserve capacity and WFEBC links values for Net3. The reserve capacity of the individual links of Net3 is plotted against their WFEBC value

Figures 2 and 3 show the criticality of exclusive supply paths (forest links and bridges) according to both metrics. The analysis of the reserve capacity and WFEBC values of the other links shows:

- Excluding the forest and bridges of the network, the individual failures of 16 links of Net3 (represented in blue or green in Fig. 3) cause the reserve capacity of the network to drop below 1, based on the hydraulic CLA. The graph theoretic analysis of Net3 identifies the same 16 links as having high WFEBC values, which indicates that these links contribute significantly to the supply of the network.
- The remaining links (represented in yellow or orange in Fig. 3) have indiscernibly high values of reserve capacity, their individual failures still allowing the network to meet up to 190% of the nominal demand at all nodes. High values of reserve capacity mostly coincide with low WFEBC values (see Fig. 4), except for a few exceptions. Among the high reserve capacity links, some also display relatively high WFEBC values. Biased by the definition of a critical point, the high reserve capacities associated with the failure of these pipes fail to highlight their critical local contribution to the supply of Net3. This is however reflected by their high WFEBC values.

Figure 4 presents the correlation between WFEBC and reserve capacity in the analysis of Net3. It shows how WFEBC differs from the reserve capacity and flags all locally critical links, unbiased by the definition of a critical point. The properties of WFEBC make it an adequate pre-selection tool of critical links for further hydraulic analysis.

### BWFL

The comparative analysis of the hydraulic and graph theoretic resilience assessment methods is further extended by applying the two methods to a complex operational network. BWFL is a water supply network located in the UK, and consisting of 2312 nodes and 2369 links; this network comprises around 20 times more links and nodes than Net3.

BWFL was originally sectorised into two single feed DMAs (by means of two boundary valves, *BV*_1_ and *BV*_2_) and one cascading DMA. In 2012, BWFL was upgraded with dynamically adaptive DMA boundaries. Water distribution networks with dynamically adaptive topology is a novel operational approach that was introduced to improve the resilience, pressure management and water quality of sectorised WDNs (Wright et al. 2014; Wright et al. 2015). DMAs are dynamically aggregated during the day into large multi-feed pressure managed areas, and automatically sectorised at night for leakage detection and monitoring (Wright et al. 2014). For this case study, WFEBC is applied to the sectorised (disconnected) topology of BWFL and benchmarked against a hydraulic model based analysis.

Criticality analyses have been previously carried out for BWFL. Herrera et al. (2015) proposed a graph theoretic metric for the resilience analysis of the network and Wright et al. (2015) carried out a hydraulic CLA limited to a few candidate links to solve an optimal valve placement problem. The work presented in this paper extends the restricted hydraulic CLA carried out by Wright et al. (2015) to the whole network using the reserve capacity as a measure for link criticality. The hydraulic model based analysis is used to benchmark the performance of WFEBC.

Several differences between BWFL and Net3 impact the presented resilience analysis. BWFL is significantly larger than Net3 and it differs from the latter with respect to pipe roughness and spatial homogeneity. It is thus necessary to make a few preliminary observations and modifications before the criticality assessment of BWFL is discussed.

**Fig. 5:**
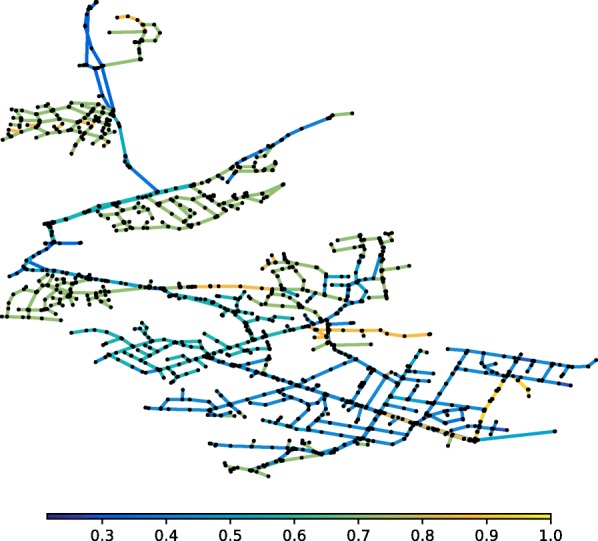
Analysis of the roughness of BWFL pipes. The colour of the links represent the normalised roughness coefficient of the pipes. Compared to Net3, BWFL is geographically heterogenous with regards to pipe roughness

**Pipe roughness** For the graph theoretic analysis of Net3, the random walks are computed through the network links weighed by resistance coefficients accounting only for the lengths and diameters of the pipes. Despite neglecting the roughness coefficients of the pipes, the results proved satisfactory in comparison with the hydraulic analysis. A posteriori, this can be explained by the homogeneity of roughness coefficients across Net3. Figure 5 represents the normalised roughness coefficients of BWFL pipes: the greater the roughness coefficient of a pipe, the greater the resistance of the link. BWFL presents significant local variations in pipe roughness that justify that roughness coefficients be accounted for in the link resistance weights to ensure the analysis yields optimal results. The weighted adjacency matrix of the network (Eq. 2) is edited in accordance with the Darcy-Weisbach equation, which relates the head loss, or pressure loss, due to friction along a given length of pipe to the average velocity of the fluid flow, in order to incorporate pipe roughness into link conductance coefficients:

9 $$ A_{ij} = \left\{\begin{array}{ll} \frac{d_{ij}}{L_{ij}\epsilon_{ij}} & \text{if there is an edge e connecting node i and j,}\\ 0 & \text{otherwise.} \end{array}\right.   $$

where *L*_*ij*_, *d*_*ij*_ and *ε*_*ij*_ are respectively the length, diameter and roughness coefficient of the pipe connecting nodes i and j. WFEBC is computed for all network links using the new weighted adjacency matrix defined by Eq. 9 instead of 2.

**Forest-core decomposition** The computational time for the resilience analysis increases rapidly with the size of a network. As a result, the analysis of BWFL took several hours to run, in comparison to minutes for Net3. The computational time was significantly reduced by restricting the analysis to a smaller subset of links of the original network. This was achieved by carrying out a preliminary model reduction step.

Model reduction procedures developed for WDNs (Deuerlein 2008) have been applied to improve the computational performance of hydraulic solvers for large scale systems (Deuerlein et al. 2015; Simpson et al. 2012). Model decomposition allows to clearly represent the interactions between different supply zones and better define optimisation problems (Di Nardo and Di Natale 2011). The decomposition method relies on the identification of two complementary components of the network: the forest of sub-trees and the simple core (or *two-core*) of the network. The simple core of the network is obtained by recursively removing all nodes with degrees strictly smaller than 2 (also called *leaves* of the network) along with the parent edge connecting them to their associated *root* node. The remaining model features biconnected blocks connected by bridge elements, which make up the simple core. The complementary links and nodes to the simple core constitute the forest of the network. For more details about the graph decomposition procedure, the reader is referred to (Deuerlein 2008). To ensure the resilience analysis of the reduced model is equivalent to that of the all mains model, the demands of the simple core root nodes must be updated as the network leaves are pruned off: every time a leaf node is removed from the network, its demand is added onto the demand of its root node. This is sufficient to ensure the graph theoretic analysis of the reduced model is equivalent to that of the all-mains model. The hydraulic resilience analysis of the skeletonised model, on the other hand, must be run on the full model, as the reserve capacity must account for the head losses along forest connections. The effects of individual failure of network forest links can be assessed separately from the rest of the network. Their identification can be supported by graph decomposition (Deuerlein 2008).

**Fig. 6:**
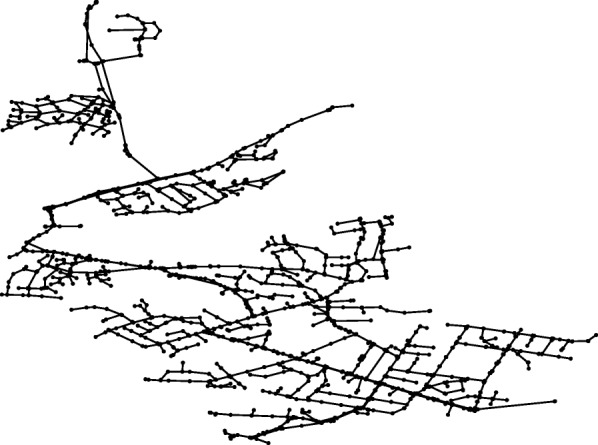
All mains model of BWFL. The all mains model of BWFL represents the network before the reduction step is undertaken

**Fig. 7:**
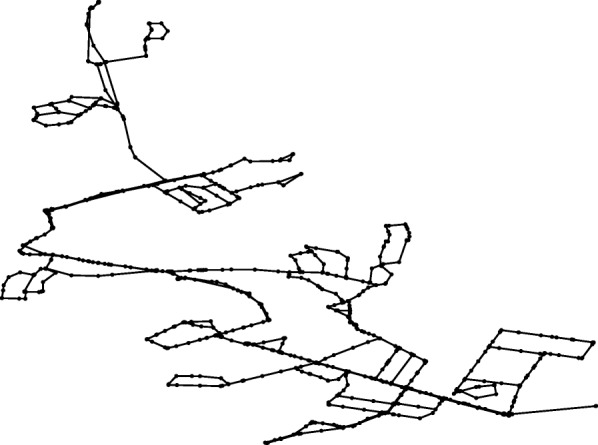
Core model of BWFL. The core model of BWFL represents the network after removal of the forest links

The developed computational procedure was applied to the original all mains model of BWFL. The forest-core decomposition reduces the network model by 1177 pipes and 1176 nodes, pruning off about half of all network components (49.68% of the links and 50.91% of the nodes). The reduced network model is represented against the all mains model in Figs. 6 and 7. Consequently, the graph theoretic analysis of the simple core of BWFL is done within 30 min. The hydraulic criticality analysis on the other hand takes over 30 h to run. It is worth noting that both the graph theoretic and hydraulic analyses of BWFL are significantly more time consuming than for Net3, which shows that WFEBC too is affected, to a smaller extent, by computational limitations for assessing resilience for large scale operational networks.

**Fig. 8:**
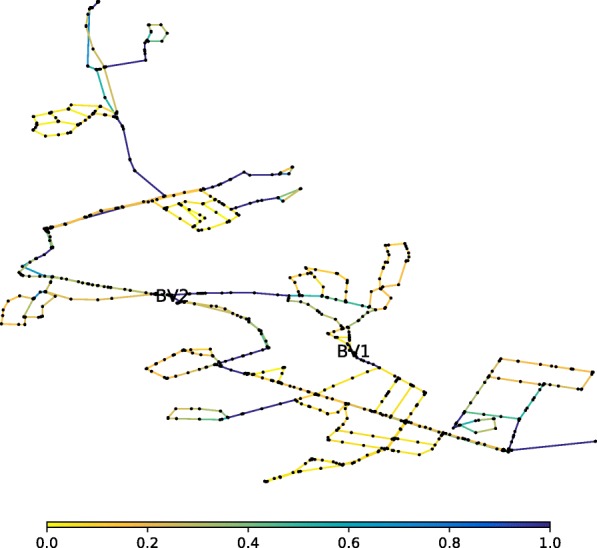
WFEBC of BWFL core links. After the forest-core decomposition is applied, only the links of the core model of BWFL are analysed. The darker the link, the more critical

**Fig. 9:**
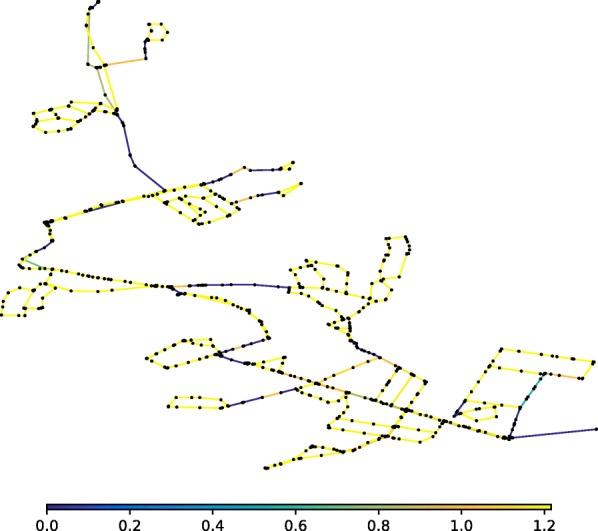
Reserve capacity of BWFL core links. Only the reserve capacity of BWFL core links is computed. The hydraulic model based analysis still requires to be run on the full model, to account for head losses in the forest links. The darker the link, the more critical

WFEBC values are comprised between 0 and 1. The smaller the WFEBC of a link, the less critical it is. Bridges (or bottlenecks) on the other hand, are exclusive supply links, associated with a WFEBC value of 1. The reserve capacity of a link represents the maximum fraction of total nominal demand the network can supply if the link has failed. In the case of BWFL, reserve capacity values extend from 0 (i.e. one or more nodes cannot be supplied following the failure of the link) for bridges to approximately 1.36 (i.e. all nodes can be supplied at least 136% of their nominal capacity at all times) for the least critical links. The results of the graph theoretic WFEBC analysis and hydraulic model based CLA of BWFL links are represented in Figs. 8 and 9: the less critical the link, the closer its colour is to yellow on the selected colour scale; more critical links are represented in dark blue. We observe that:

- Most low reserve capacity links coincide with high WFEBC links. These observations concur with the conclusions of the comparative analysis for Net3. The correlation between WFEBC and reserve capacity is sufficiently good to envisage the application of the surrogate measure for the identification and prioritisation of critical pipes (or areas) for further hydraulic analysis (see Fig. 11);

**Fig. 10:**
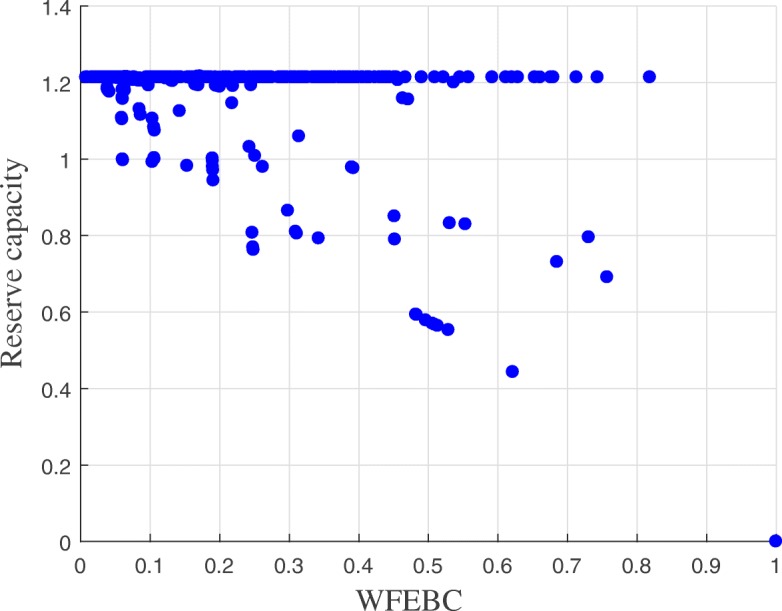
Analysis of the correlation between reserve capacity and WFEBC links values for BWFL. The reserve capacity of the individual links of the core model of BWFL is plotted against their WFEBC value

**Fig. 11:**
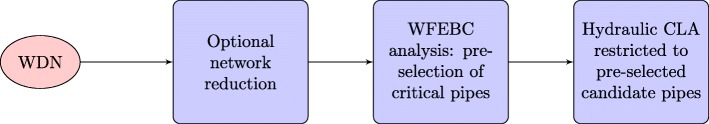
Hybrid critical link analysis. The flowchart illustrates a hybrid methodology for critical link analysis: following an optional network reduction step, a restricted set of critical pipes is pre-selected based on their WFEBC values for further hydraulic analysis (i.e. implementing reserve capacity). This is made possible by the relation between WFEBC and reserve capacity values (see Figure 10)
- As for Net3, the results of the graph theoretic and hydraulic analyses of BWFL reveal some discrepancies. If links exhibiting reserve capacity values greater than 1 are mainly associated with lower WFEBC values, they also are, in some cases, associated to links with relatively high WFEBC. The high local contribution of these links is not reflected by their associated reserve capacity, as the nodes they supply are closer, or easier to supply, than the critical point of the network. As a result, reserve capacity cannot accurately reflect the true criticality of the network links, as it relies on the definition of a critical point which might not hold under failure conditions (e.g. significant change in local demand).

The results of the correlation analysis are summed up by the correlation map presented in Fig. 10, which represents the reserve capacity of the links of the reduced BWFL model plotted against their WFEBC values. It emerges from the analysis of BWFL that the criticality measure reserve capacity is biased; it sometimes falls short of identifying pipe failures leading to significant head losses if the supply of the critical node is not affected. As a result, reserve capacity fails to assess the global impact of pipe failures on the network. On the other hand, WFEBC better reflects the relative contribution of alternative supply paths both to the global and local topological and energy redundancy of the network. The properties of the surrogate measure also make it an adequate pre-selection tool of critical links for further analysis, as illustrated by the flowchart in Fig. 11. Figure 10 shows that a fraction of pipes with reserve capacity greater than 1 can be filtered out prior to the hydraulic CLA based on their WFEBC values (smaller than 1.17 approximately) with low chances of eliminating critical pipes in the process, as lower WFEBC values are systematically associated with high reserve capacity values.

It is important to notice that neither the WFEBC nor the reserve capacity values of the BWFL links seem to show a correlation with the diameter or conductance coefficients (*A*_*ij*_) of the pipes, suggesting the criticality of individual pipes is not defined so much by their physical characteristics as by their position in the network and the availability of alternative paths.

**Fig. 12:**
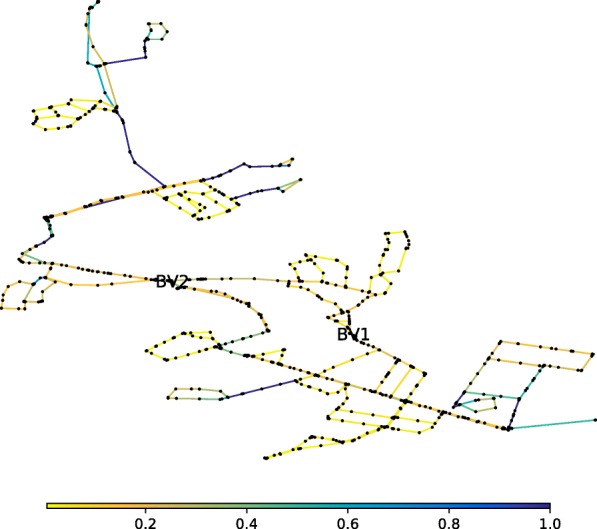
WFEBC of BWFL core model links, dynamically adaptive topology. Boundary valves *BV*_1_ and *BV*_2_ are opened to assess the resilience of the paired DMAs

The analysis of BWFL also shows how specific topological features influence the resilience of the network. In the previous analysis, BWFL is a disconnected graph: the boundary valves located in *BV*_1_ and *BV*_2_ allow to close off the links bridging the upper and lower areas of the network and split the network into two connected components (or DMAs). This is reflected in the resilience analysis of BWFL, which shows high concentration of flow in single supply paths and poor customer reachability in parts of the network, as each DMA is effectively supplied by a single source (see Fig. 8). The permanent closure of boundary valves has numerous drawbacks, including higher energy losses across the network, water quality issues, due to the presence of dead ends, and reduced resilience to failure (Wright et al. 2015). To illustrate the impact of sectorisation on the resilience of the network, *BV*_1_ and *BV*_2_ are opened to combine (or pair) the two single-feed DMAs into a large multi-feed pressure managed area (paired configuration), and a new resilience analysis is conducted. Figure 12 represents the WFEBC of BWFL links in the paired (connected) configuration. As before, values are comprised between 0 and 1 and lower value (yellow links) correspond to less critical pipes when values closer to 1 (dark blue links) correspond to exclusive supply pipes.

**Fig. 13:**
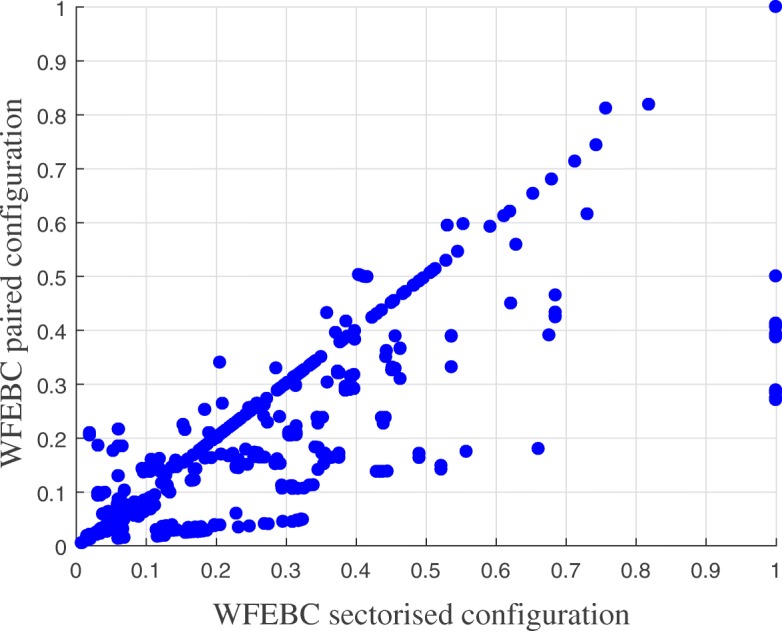
Comparison of link WFEBC values in the paired DMA configuration of BWFL against the sectorised configuration. The average decrease in FEBC indicates an improvement with regards to resilience in the paired configuration compared to the sectorised topology

The results are compared to Fig. 8 (sectorised or disconnected configuration). Lower average WFEBC values for the dynamically adaptive DMAs (paired configuration) compared to the sectorised network topology (see Fig. 13) demonstrate better distribution of flow among alternative supply paths and fewer single supply paths. The dynamically adaptive topology also increases the source redundancy and reachability of customer nodes (Wright et al. 2014). The results of the analysis demonstrate how the resilience of a network with dynamically adaptive topology can benefit from improved redundancy and control. The current approach only relies on targeted DMA pairing and allows to increase the resilience of a network at minimum cost.

## Conclusions

The resilience of water distribution networks is achieved through resilient design and the implementation of monitoring and control capabilities to support the detection, response and recovery from disruptive events. Water utilities are expected to build resilience within the water distribution infrastructure in order to provide continuity of services to its customers in the face of asset deterioration and growing environmental and man-made threats. However, with such a complex and interconnected network, it is difficult to accurately identify and understand critical links that could lead to disruption of services.

Following a review of existing measures of network resilience and component criticality, this paper presents a measure of link (pipe) criticality for the resilience analysis of WDNs. The measure combines graph-theoretic methods with pipe hydraulics principles: random walk betweenness centrality is complemented with hydraulic energy loss principles in pipes in order to derive a hydraulically informed graph theoretic measure of link criticality. The proposed measure is applied to two case study networks, Net3 and BWFL. The analysis also implements network decomposition in order to facilitate the application of the approach to complex operational networks. The results are compared to a traditional hydraulic model based critical link analysis. It is important to notice that both Net3 and BWFL present a near tree layout, with average node degrees of respectively 2.51 and 2.05. Further research is required to investigate the effect of the near tree layout on the outcome of the analysis and determine the accuracy of the analysis on more looped networks.

The analysis of the case studies reveal both important similarities and differences between the proposed hydraulically informed graph theoretic method (WFEBC) and the benchmark hydraulic model based method (reserve capacity). First, the results from Net3 and BWFL demonstrate that WFEBC can be applied to identify links and network structures that are critical for the resilience of a water distribution network. Secondly, the hydraulically informed graph theoretic CLA and following resilience analysis of BWFL demonstrate that WFEBC can be applied to benchmark the redundancy of network topologies and facilitate the design of dynamically adaptive DMAs (Wright et al. 2014; Wright et al. 2015). Thirdly, the computational time for the hydraulically informed graph theoretic measure was nearly two orders of magnitude faster than the hydraulic model based CLA for the complex operational water distribution network BWFL (e.g. 30 min versus 30 h). Finally, the noted discrepancies in the CLAs are attributed to fundamental differences between WFEBC and reserve capacity. The reserve capacity is susceptible to a critical point bias in comparison to WFEBC. This allows WFEBC to identify alternative paths that mostly contribute to the local and global resilience of supply for a network. The hydraulically informed graph theoretic measure, however, cannot accurately model the hydraulic behaviour of a WDN or assess the extraordinary demand the network is able to meet.

Given the breadth in activity and operational scenarios that the term resilience covers, it is unlikely that it can be reflected in a single metric. The presented analysis indicates that the proposed hydraulically informed surrogate measure of pipe criticality, Water Flow Edge Betweenness Centrality, can successfully complement a hydraulic model based method for estimating the criticality of individual links if an accurate hydraulic model of a network exists. In this case, a multi-stage approach can be envisaged: a preliminary ranking of critical links based on WFEBC would be followed by a detailed hydraulic model based analysis with the simulation of failure scenarios.

Further work is required to apply WFEBC to a range of operational networks in order to validate its applicability and robustness; and also explore an optimal hybrid approach that integrates the proposed hydraulically informed graph theoretic measure of link criticality with hydraulic model based methods for the resilience analysis of water distribution networks.

## Abbreviations

BV: Boundary valve
BWFL: Bristol water field lab
CLA: Critical link analysis
DDA: Demand driven analysis
DMA: District metered area
PDA: Pressure driven analysis
RBC: Routing betweenness centrality
WDN: Water distribution network
WFEBC: Water flow edge betweenness centrality
